# 2,4-Dibromo-1,3-dimeth­oxy-5-methyl­benzene

**DOI:** 10.1107/S1600536812025767

**Published:** 2012-06-13

**Authors:** Sunayna Pawar, Mahidansha Shaikh, Neil Koorbanally, Bernard Omondi, Deresh Ramjugernath

**Affiliations:** a School of Chemistry and Physics, University of KwaZulu-Natal, Westville Campus, Private Bag X54001, Durban 4000, South Africa; b School of Engineering, University of KwaZulu-Natal, Howard College Campus, Private Bag X54001, Durban, 4000, South Africa

## Abstract

The title compound, C_9_H_10_Br_2_O_2_, crystallizes with two mol­ecules in the asymmetric unit. The two mol­ecules are essentially planar with slight differences in the (Br)C—C—O—C(H_3_) torsion angles [−176.7 (2) and −172.8 (2)° in one mol­ecule and 174.8 (2) and 179.9 (2)° in the other]. The crystal structure consists of sheets of mol­ecules linked through Br⋯Br [3.3547 (4), 3.3703 (4) and 3.5379 (4) Å] inter­actions, which are in turn connected through π–π inter­actions with centroid–centroid distances of 3.5902 (14) and 3.5956 (14) Å.

## Related literature

For related structures, see: Hernandez *et al.* (2003 ▶); Cukiernik *et al.* (2008 ▶); Saeed *et al.* (2010 ▶); Koorbanally *et al.* (2004 ▶). For bond-length data, see: Allen *et al.* (1987 ▶).
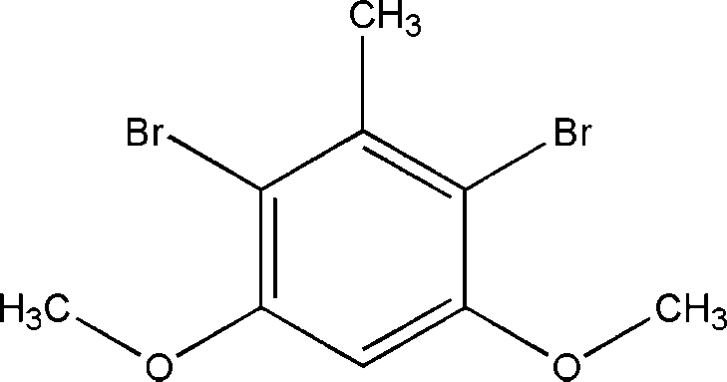


## Experimental

### 

#### Crystal data

C_9_H_10_Br_2_O_2_
*M* *_r_* = 309.99
Monoclinic, 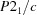

*a* = 8.7653 (2) Å
*b* = 16.4434 (3) Å
*c* = 13.8895 (3) Å
β = 91.715 (1)°
*V* = 2001.02 (7) Å^3^
*Z* = 8
Mo *K*α radiation
μ = 8.07 mm^−1^
*T* = 446 K
0.55 × 0.25 × 0.10 mm

#### Data collection

Bruker SMART APEXII CCD diffractometer
Absorption correction: multi-scan (*SADABS*; Bruker, 2008 ▶) *T*_min_ = 0.095, *T*_max_ = 0.499
36582 measured reflections
4979 independent reflections
4447 reflections with *I* > 2σ(*I*)
*R*_int_ = 0.037

#### Refinement

*R*[*F* ^2^ > 2σ(*F* ^2^)] = 0.027
*wR*(*F* ^2^) = 0.076
*S* = 1.04
4979 reflections
241 parameters
H-atom parameters constrained
Δρ_max_ = 0.79 e Å^−3^
Δρ_min_ = −0.69 e Å^−3^

### 

Data collection: *APEX2* (Bruker, 2008 ▶); cell refinement: *SAINT-Plus* (Bruker, 2008 ▶); data reduction: *SAINT-Plus* and *XPREP* (Bruker, 2008 ▶); program(s) used to solve structure: *SHELXS97* (Sheldrick, 2008 ▶); program(s) used to refine structure: *SHELXL97* (Sheldrick, 2008 ▶); molecular graphics: *ORTEP-3* (Farrugia, 1997 ▶); software used to prepare material for publication: *WinGX* (Farrugia, 1999 ▶).

## Supplementary Material

Crystal structure: contains datablock(s) I, New_Global_Publ_Block. DOI: 10.1107/S1600536812025767/fj2564sup1.cif

Structure factors: contains datablock(s) I. DOI: 10.1107/S1600536812025767/fj2564Isup2.hkl

Supplementary material file. DOI: 10.1107/S1600536812025767/fj2564Isup3.cml

Additional supplementary materials:  crystallographic information; 3D view; checkCIF report

## Figures and Tables

**Fig. 1.**
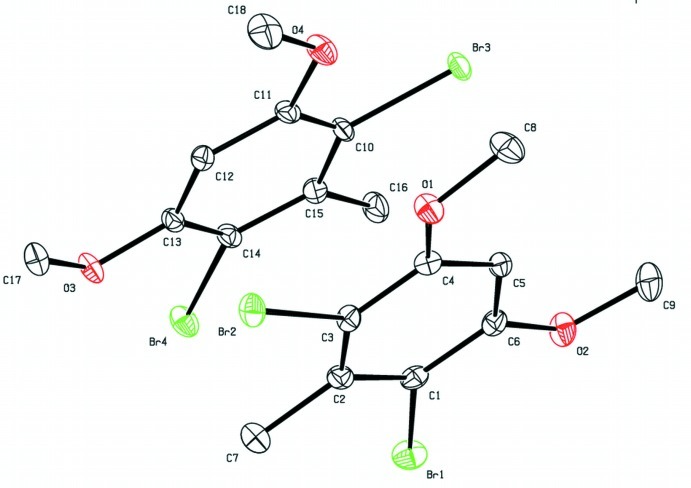
The asymmetric unit of the title compound with displacement ellipsoids drawn at the 50% probability level.

## supplementary crystallographic information

### Comment

The title compound (I), is an important precursor in the synthesis of 2,4-dihydroxy-1,3-dimethoxy-5-methylbenzene, a key intermediate in the synthesis of Drimiopsin A (Koorbanally *et al.*, 2004).

The two molecules are essentialy planar with a mean plane deviation of -0.010 (1)°, -0.018 (1)°, 0.031 (1)° and 0.003 (1)° for Br1 to Br4 respectively. All bond distances and angles are within normal ranges (Allen *et al.*, 1987) (Fig 1).

In the crystal, molecules are connected through three diferent Br···Br (Br1···Br1 = 3.3547 (4), Br2···Br2 = 3.3703 (4) and Br3···Br4 = 3.5379 (4) Å, Symmetry codes: 1 - *x*, -*y*, 2 - *z*; 1 - *x*, 1 - *y*, 2 - *z*; -1 + *x*, *y*, *z*) intermolecular interactions and a Br···O interaction (3.2657 (18) Å Symmetry code: -1 + *x*, *y*, *z*) resulting in sheets along the *ab* face. These sheets are in turn connected through π···π interactions with distances between 3.5902 (14) and 3.5956 (15) Å (Symmetry code: *x*, *y*, 1 - *z*).

### Experimental

To a solution of 1,3-dimethyl-5-methylbenzene (1.0 g, 6.578 mmol,1 eq.) in water (40 ml), bromine (3.1 ml, 39.473 mmol, 6.0 eq.) was added at 0 *^o^*C and the resultant solution refluxed at 110 *^o^*C for 12 h. The reaction was monitored by TLC using EtOAc/ Hexane (5/95, *Rf* = 0.6). After completion of the reaction, the reaction mixture was diluted with ethyl acetate (30 ml) and washed with 20 ml of water. The organic layer was separated and dried over anhydrous MgSO_4._ The solvent was evaporated under reduced pressure to afford a crude mixture of dibromo and tribromo-compound. This was purified by column chromatography using 100% hexane. Colourless crystals were obtained by slow evaporation of the solvents from solutions of the title compound in a mixture of ethyl acetate and hexane to yield the title compound with a m.p. of 165–167 °C.

^1^H NMR (400 MHz, CDCl_3_): δ (p.p.m.): 6.42 (1*H*, s), 3.90 (6*H*, s), 2.61 (3*H*, s). ^13^C NMR (100 MHz, CDCl_3_): δ (p.p.m.): 155.9, 139.4, 105.9, 95.0, 56.7, 24.3

### Refinement

All H atoms were positioned geometrically and allowed to ride on their respective parent atoms. The carboxyl H atoms were located from the difference map and allowed to ride on their parent atoms. All H atoms were refined isotropically.

### Crystal data

**Table:**

C_9_H_10_Br_2_O_2_	*F*(000) = 1200
*M**_r_* = 309.99	*D*_x_ = 2.058 Mg m^−^^3^
Monoclinic, *P*2_1_/*c*	Mo *K*α radiation, λ = 0.71073 Å
Hall symbol: -P 2ybc	Cell parameters from 37343 reflections
*a* = 8.7653 (2) Å	θ = 1.9–28.3°
*b* = 16.4434 (3) Å	µ = 8.07 mm^−^^1^
*c* = 13.8895 (3) Å	*T* = 446 K
β = 91.715 (1)°	Block, colourless
*V* = 2001.02 (7) Å^3^	0.55 × 0.25 × 0.10 mm
*Z* = 8	

### Data collection

**Table:**

Bruker SMART APEXII CCD diffractometer	4979 independent reflections
Radiation source: fine-focus sealed tube	4447 reflections with *I* > 2σ(*I*)
Graphite monochromator	*R*_int_ = 0.037
φ and ω scans	θ_max_ = 28.3°, θ_min_ = 1.9°
Absorption correction: multi-scan (*SADABS*; Bruker, 2008)	*h* = −11→11
*T*_min_ = 0.095, *T*_max_ = 0.499	*k* = −21→20
36582 measured reflections	*l* = −18→18

### Refinement

**Table:**

Refinement on *F*^2^	Primary atom site location: structure-invariant direct methods
Least-squares matrix: full	Secondary atom site location: difference Fourier map
*R*[*F*^2^ > 2σ(*F*^2^)] = 0.027	Hydrogen site location: inferred from neighbouring sites
*wR*(*F*^2^) = 0.076	H-atom parameters constrained
*S* = 1.04	*w* = 1/[σ^2^(*F*_o_^2^) + (0.0416*P*)^2^ + 3.5276*P*] where *P* = (*F*_o_^2^ + 2*F*_c_^2^)/3
4979 reflections	(Δ/σ)_max_ = 0.029
241 parameters	Δρ_max_ = 0.79 e Å^−^^3^
0 restraints	Δρ_min_ = −0.69 e Å^−^^3^

### Special details

**Table:**

Experimental. Carbon-bound H-atoms were placed in calculated positions [C—H = 0.96 Å for Me H atoms and 0.93 Å for aromatic H atoms; *U*_iso_(H) = 1.2*U*_eq_(C) (1.5 for Me groups)] and were included in the refinement in the riding model approximation.
Geometry. All e.s.d.'s (except the e.s.d. in the dihedral angle between two l.s. planes) are estimated using the full covariance matrix. The cell e.s.d.'s are taken into account individually in the estimation of e.s.d.'s in distances, angles and torsion angles; correlations between e.s.d.'s in cell parameters are only used when they are defined by crystal symmetry. An approximate (isotropic) treatment of cell e.s.d.'s is used for estimating e.s.d.'s involving l.s. planes.
Refinement. Refinement of *F*^2^ against ALL reflections. The weighted *R*-factor *wR* and goodness of fit *S* are based on *F*^2^, conventional *R*-factors *R* are based on *F*, with *F* set to zero for negative *F*^2^. The threshold expression of *F*^2^ > σ(*F*^2^) is used only for calculating *R*-factors(gt) *etc*. and is not relevant to the choice of reflections for refinement. *R*-factors based on *F*^2^ are statistically about twice as large as those based on *F*, and *R*- factors based on ALL data will be even larger.>>> The Following Model ALERTS were generated - (Acta-Mode) <<< Format: alert-number_ALERT_alert-type_alert-level text112_ALERT_2_B ADDSYM Detects Additional (Pseudo) Symm. Elem··· *Z*Author response: No additional symmetry.912_ALERT_4_C Missing # of FCF Reflections Above STh/*L*= 0.600 14 431_ALERT_2_G Short Inter HL..A Contact Br1.. Br1.. 3.35 A ng. 431_ALERT_2_G Short Inter HL..A Contact Br2.. Br2.. 3.37 A ng. 431_ALERT_2_G Short Inter HL..A Contact Br3.. Br4.. 3.54 A ng.Noted:

### Fractional atomic coordinates and isotropic or equivalent isotropic displacement parameters (Å^2^)

**Table:**

	*x*	*y*	*z*	*U*_iso_*/*U*_eq_	
C1	0.7073 (3)	0.18056 (17)	0.96286 (18)	0.0134 (5)	
C2	0.6244 (3)	0.25269 (17)	0.95874 (18)	0.0139 (5)	
C3	0.7078 (3)	0.32456 (16)	0.95817 (18)	0.0125 (5)	
C4	0.8676 (3)	0.32527 (16)	0.96239 (18)	0.0128 (5)	
C5	0.9465 (3)	0.25226 (15)	0.96624 (18)	0.0126 (5)	
H5	1.0526	0.2522	0.9683	0.015*	
C6	0.8670 (3)	0.17921 (17)	0.96708 (18)	0.0137 (5)	
C7	0.4523 (3)	0.25224 (18)	0.9553 (2)	0.0194 (6)	
H7A	0.4161	0.2323	0.8938	0.029*	
H7B	0.4153	0.3065	0.9647	0.029*	
H7C	0.4160	0.2176	1.0053	0.029*	
C8	1.1004 (3)	0.4005 (2)	0.9614 (2)	0.0225 (6)	
H8A	1.1412	0.3740	1.0181	0.034*	
H8B	1.1352	0.4558	0.9602	0.034*	
H8C	1.1342	0.3725	0.9052	0.034*	
C9	1.0984 (3)	0.10374 (19)	0.9875 (2)	0.0219 (6)	
H9A	1.1471	0.1228	0.9308	0.033*	
H9B	1.1310	0.0491	1.0014	0.033*	
H9C	1.1259	0.1384	1.0409	0.033*	
C10	0.8338 (3)	0.23502 (18)	0.71339 (18)	0.0139 (5)	
C11	0.7952 (3)	0.31760 (18)	0.71368 (18)	0.0138 (5)	
C12	0.6418 (3)	0.33975 (17)	0.71153 (18)	0.0131 (5)	
H12	0.6145	0.3944	0.7121	0.016*	
C13	0.5294 (3)	0.28001 (17)	0.70859 (18)	0.0125 (5)	
C14	0.5708 (3)	0.19796 (17)	0.70738 (18)	0.0129 (5)	
C15	0.7239 (3)	0.17377 (17)	0.70967 (18)	0.0134 (5)	
C16	0.7691 (3)	0.08609 (18)	0.7087 (2)	0.0186 (6)	
H16A	0.8527	0.0788	0.6664	0.028*	
H16B	0.6840	0.0538	0.6864	0.028*	
H16C	0.7997	0.0694	0.7726	0.028*	
C17	0.3326 (3)	0.38005 (18)	0.7161 (2)	0.0190 (6)	
H17A	0.3769	0.4020	0.7746	0.028*	
H17B	0.2234	0.3834	0.7180	0.028*	
H17C	0.3676	0.4106	0.6622	0.028*	
C18	0.8728 (4)	0.45640 (19)	0.7160 (2)	0.0241 (6)	
H18A	0.8046	0.4679	0.6624	0.036*	
H18B	0.9638	0.4885	0.7109	0.036*	
H18C	0.8238	0.4695	0.7750	0.036*	
O1	0.9371 (2)	0.39911 (12)	0.96234 (15)	0.0170 (4)	
O2	0.9358 (2)	0.10511 (12)	0.97172 (15)	0.0180 (4)	
O3	0.3772 (2)	0.29668 (12)	0.70659 (14)	0.0162 (4)	
O4	0.9121 (2)	0.37173 (12)	0.71589 (15)	0.0189 (4)	
Br1	0.60437 (3)	0.078949 (17)	0.96307 (2)	0.01967 (8)	
Br2	0.60863 (3)	0.427111 (17)	0.95128 (2)	0.01804 (8)	
Br3	1.04452 (3)	0.208463 (18)	0.718591 (18)	0.01701 (8)	
Br4	0.41110 (3)	0.120197 (17)	0.70330 (2)	0.01769 (8)	

### Atomic displacement parameters (Å^2^)

**Table:**

	*U*^11^	*U*^22^	*U*^33^	*U*^12^	*U*^13^	*U*^23^
C1	0.0170 (13)	0.0102 (12)	0.0129 (11)	−0.0039 (10)	0.0000 (9)	0.0017 (9)
C2	0.0138 (12)	0.0178 (14)	0.0103 (11)	−0.0007 (10)	0.0013 (9)	−0.0017 (10)
C3	0.0153 (12)	0.0088 (12)	0.0134 (11)	0.0039 (9)	0.0009 (9)	−0.0014 (9)
C4	0.0161 (12)	0.0104 (12)	0.0121 (11)	−0.0012 (10)	0.0013 (9)	−0.0015 (9)
C5	0.0123 (12)	0.0122 (13)	0.0134 (11)	0.0019 (9)	0.0007 (9)	0.0004 (9)
C6	0.0168 (12)	0.0121 (13)	0.0123 (11)	0.0020 (10)	0.0000 (9)	0.0005 (10)
C7	0.0144 (13)	0.0213 (16)	0.0224 (14)	0.0007 (11)	0.0020 (10)	−0.0010 (11)
C8	0.0170 (14)	0.0199 (15)	0.0308 (16)	−0.0039 (11)	0.0042 (12)	−0.0013 (12)
C9	0.0206 (14)	0.0145 (14)	0.0302 (15)	0.0060 (11)	−0.0040 (11)	−0.0006 (12)
C10	0.0070 (11)	0.0224 (14)	0.0122 (11)	0.0004 (10)	0.0005 (9)	−0.0001 (10)
C11	0.0098 (11)	0.0187 (14)	0.0129 (11)	−0.0046 (10)	0.0001 (9)	0.0003 (10)
C12	0.0126 (12)	0.0126 (13)	0.0141 (11)	0.0012 (10)	−0.0001 (9)	0.0014 (10)
C13	0.0099 (11)	0.0166 (13)	0.0111 (11)	0.0013 (10)	0.0010 (9)	−0.0016 (10)
C14	0.0097 (11)	0.0147 (13)	0.0141 (11)	−0.0028 (10)	0.0003 (9)	−0.0008 (10)
C15	0.0130 (12)	0.0158 (13)	0.0113 (11)	0.0014 (10)	0.0002 (9)	−0.0016 (10)
C16	0.0147 (13)	0.0153 (14)	0.0259 (14)	0.0041 (10)	0.0019 (10)	0.0023 (11)
C17	0.0132 (12)	0.0210 (15)	0.0226 (13)	0.0035 (11)	−0.0014 (10)	−0.0012 (11)
C18	0.0200 (14)	0.0161 (15)	0.0362 (17)	−0.0078 (12)	−0.0010 (12)	0.0002 (13)
O1	0.0148 (9)	0.0095 (9)	0.0269 (10)	−0.0020 (7)	0.0015 (8)	0.0001 (8)
O2	0.0180 (10)	0.0090 (9)	0.0268 (10)	0.0020 (8)	−0.0022 (8)	0.0012 (8)
O3	0.0084 (8)	0.0163 (10)	0.0239 (10)	0.0015 (7)	0.0012 (7)	−0.0024 (8)
O4	0.0127 (9)	0.0163 (11)	0.0277 (10)	−0.0044 (8)	0.0015 (8)	−0.0017 (8)
Br1	0.02245 (15)	0.01443 (15)	0.02218 (14)	−0.00754 (10)	0.00115 (11)	−0.00034 (10)
Br2	0.01823 (14)	0.01405 (14)	0.02181 (14)	0.00654 (10)	−0.00010 (10)	−0.00210 (10)
Br3	0.00848 (12)	0.02530 (16)	0.01724 (13)	0.00245 (10)	0.00042 (9)	−0.00052 (10)
Br4	0.01199 (13)	0.01491 (14)	0.02615 (15)	−0.00285 (10)	0.00015 (10)	−0.00184 (10)

### Geometric parameters (Å, º)

**Table:**

C1—C2	1.391 (4)	C10—C15	1.394 (4)
C1—C6	1.400 (4)	C10—C11	1.399 (4)
C1—Br1	1.899 (3)	C10—Br3	1.897 (3)
C2—C3	1.390 (4)	C11—O4	1.357 (3)
C2—C7	1.508 (4)	C11—C12	1.392 (4)
C3—C4	1.400 (4)	C12—C13	1.391 (4)
C3—Br2	1.898 (3)	C12—H12	0.9300
C4—O1	1.359 (3)	C13—O3	1.361 (3)
C4—C5	1.386 (4)	C13—C14	1.397 (4)
C5—C6	1.389 (4)	C14—C15	1.400 (4)
C5—H5	0.9300	C14—Br4	1.895 (3)
C6—O2	1.360 (3)	C15—C16	1.495 (4)
C7—H7A	0.9600	C16—H16A	0.9600
C7—H7B	0.9600	C16—H16B	0.9600
C7—H7C	0.9600	C16—H16C	0.9600
C8—O1	1.431 (3)	C17—O3	1.433 (4)
C8—H8A	0.9600	C17—H17A	0.9600
C8—H8B	0.9600	C17—H17B	0.9600
C8—H8C	0.9600	C17—H17C	0.9600
C9—O2	1.436 (3)	C18—O4	1.434 (4)
C9—H9A	0.9600	C18—H18A	0.9600
C9—H9B	0.9600	C18—H18B	0.9600
C9—H9C	0.9600	C18—H18C	0.9600
			
C2—C1—C6	122.4 (2)	C11—C10—Br3	117.3 (2)
C2—C1—Br1	120.2 (2)	O4—C11—C12	123.8 (3)
C6—C1—Br1	117.4 (2)	O4—C11—C10	117.0 (2)
C3—C2—C1	116.8 (2)	C12—C11—C10	119.2 (2)
C3—C2—C7	122.0 (2)	C13—C12—C11	119.9 (3)
C1—C2—C7	121.2 (2)	C13—C12—H12	120.0
C2—C3—C4	122.2 (2)	C11—C12—H12	120.0
C2—C3—Br2	121.0 (2)	O3—C13—C12	123.4 (2)
C4—C3—Br2	116.8 (2)	O3—C13—C14	116.7 (2)
O1—C4—C5	123.4 (2)	C12—C13—C14	119.9 (2)
O1—C4—C3	117.1 (2)	C13—C14—C15	121.5 (2)
C5—C4—C3	119.5 (2)	C13—C14—Br4	117.40 (19)
C4—C5—C6	120.0 (3)	C15—C14—Br4	121.1 (2)
C4—C5—H5	120.0	C10—C15—C14	117.2 (3)
C6—C5—H5	120.0	C10—C15—C16	120.9 (2)
O2—C6—C5	123.6 (2)	C14—C15—C16	121.9 (2)
O2—C6—C1	117.2 (2)	C15—C16—H16A	109.5
C5—C6—C1	119.2 (2)	C15—C16—H16B	109.5
C2—C7—H7A	109.5	H16A—C16—H16B	109.5
C2—C7—H7B	109.5	C15—C16—H16C	109.5
H7A—C7—H7B	109.5	H16A—C16—H16C	109.5
C2—C7—H7C	109.5	H16B—C16—H16C	109.5
H7A—C7—H7C	109.5	O3—C17—H17A	109.5
H7B—C7—H7C	109.5	O3—C17—H17B	109.5
O1—C8—H8A	109.5	H17A—C17—H17B	109.5
O1—C8—H8B	109.5	O3—C17—H17C	109.5
H8A—C8—H8B	109.5	H17A—C17—H17C	109.5
O1—C8—H8C	109.5	H17B—C17—H17C	109.5
H8A—C8—H8C	109.5	O4—C18—H18A	109.5
H8B—C8—H8C	109.5	O4—C18—H18B	109.5
O2—C9—H9A	109.5	H18A—C18—H18B	109.5
O2—C9—H9B	109.5	O4—C18—H18C	109.5
H9A—C9—H9B	109.5	H18A—C18—H18C	109.5
O2—C9—H9C	109.5	H18B—C18—H18C	109.5
H9A—C9—H9C	109.5	C4—O1—C8	117.5 (2)
H9B—C9—H9C	109.5	C6—O2—C9	117.2 (2)
C15—C10—C11	122.3 (2)	C13—O3—C17	117.4 (2)
C15—C10—Br3	120.4 (2)	C11—O4—C18	117.1 (2)
			
C6—C1—C2—C3	0.5 (4)	O4—C11—C12—C13	179.5 (2)
Br1—C1—C2—C3	−179.56 (18)	C10—C11—C12—C13	−0.4 (4)
C6—C1—C2—C7	−179.3 (2)	C11—C12—C13—O3	179.8 (2)
Br1—C1—C2—C7	0.6 (3)	C11—C12—C13—C14	−0.3 (4)
C1—C2—C3—C4	−0.6 (4)	O3—C13—C14—C15	−179.7 (2)
C7—C2—C3—C4	179.3 (2)	C12—C13—C14—C15	0.4 (4)
C1—C2—C3—Br2	179.30 (18)	O3—C13—C14—Br4	−0.1 (3)
C7—C2—C3—Br2	−0.9 (3)	C12—C13—C14—Br4	−179.97 (19)
C2—C3—C4—O1	−179.4 (2)	C11—C10—C15—C14	−0.8 (4)
Br2—C3—C4—O1	0.8 (3)	Br3—C10—C15—C14	179.08 (18)
C2—C3—C4—C5	0.7 (4)	C11—C10—C15—C16	179.5 (2)
Br2—C3—C4—C5	−179.16 (19)	Br3—C10—C15—C16	−0.6 (3)
O1—C4—C5—C6	179.3 (2)	C13—C14—C15—C10	0.1 (4)
C3—C4—C5—C6	−0.8 (4)	Br4—C14—C15—C10	−179.50 (18)
C4—C5—C6—O2	−179.3 (2)	C13—C14—C15—C16	179.8 (2)
C4—C5—C6—C1	0.7 (4)	Br4—C14—C15—C16	0.2 (3)
C2—C1—C6—O2	179.4 (2)	C5—C4—O1—C8	3.2 (4)
Br1—C1—C6—O2	−0.5 (3)	C3—C4—O1—C8	−176.7 (2)
C2—C1—C6—C5	−0.6 (4)	C5—C6—O2—C9	7.2 (4)
Br1—C1—C6—C5	179.45 (19)	C1—C6—O2—C9	−172.8 (2)
C15—C10—C11—O4	−179.0 (2)	C12—C13—O3—C17	−5.3 (4)
Br3—C10—C11—O4	1.2 (3)	C14—C13—O3—C17	174.8 (2)
C15—C10—C11—C12	0.9 (4)	C12—C11—O4—C18	0.0 (4)
Br3—C10—C11—C12	−178.96 (19)	C10—C11—O4—C18	179.9 (2)
